# Incidentally Diagnosed Alport Syndrome in a Patient with Drug-Induced Vasculitis

**DOI:** 10.1155/2019/8720837

**Published:** 2019-04-10

**Authors:** D. Lai, N. Dave, R. Raghavan

**Affiliations:** ^1^Baylor College of Medicine, Houston, TX 77030, USA; ^2^Department of Medicine, Baylor College of Medicine, Houston, TX 77030, USA; ^3^Selzman Institute for Kidney Health, Baylor College of Medicine, Houston, TX 77030, USA

## Abstract

A 53-year-old woman is admitted with a serum creatinine of 16 mg/dl. Seven months earlier, she was diagnosed with heart failure and started on several medications, including Hydralazine. Laboratory studies revealed the presence of dual Anti-Neutrophil Cytoplasmic Antibodies (anti-MPO and anti-PR3), anti-nuclear and anti-histone antibodies. The clinical diagnosis was Drug-Induced ANCA Vasculitis (DIAV). Kidney histology, however, did not reveal crescents, but showed characteristic features of Alport's syndrome.

## 1. Clinical History and Initial Laboratory Data

A 53-year-old woman with Stage 3 Chronic Kidney Disease (CKD) and a baseline serum creatinine of 2.3mg/dl presented to the hospital with 2 weeks of generalized malaise and vomiting. Her past medical history was significant for hypertension, heart failure, and paroxysmal atrial fibrillation. Seven months prior to this presentation, the patient was started on carvedilol, Hydralazine, isosorbide mononitrate, and furosemide, to treat her cardiac disease. Prior to this she had not had consistent follow-up with a physician. At presentation, she reported 100% compliance with these medications. Her family history is significant for end-stage kidney disease (ESKD) in her 38-year-old brother, diagnosed 2 years earlier, of unknown etiology.

Laboratory values on presentation are listed in Table 1. Most notably, the serum creatinine was 16.6 mg/dl and there was active urinary sediment. Laboratory findings were concerning for rapidly progressive glomerulonephritis, and the history suggested a drug-induced etiology. Hydralazine was the suspected culprit and was immediately discontinued. The patient was started on pulse dose corticosteroid therapy. A noncuffed temporary dialysis catheter was placed. The patient received two hemodialysis treatments, one therapeutic plasma exchange (PLEX), and these interventions were followed by a kidney biopsy to confirm the diagnosis.

**Figure 1:**
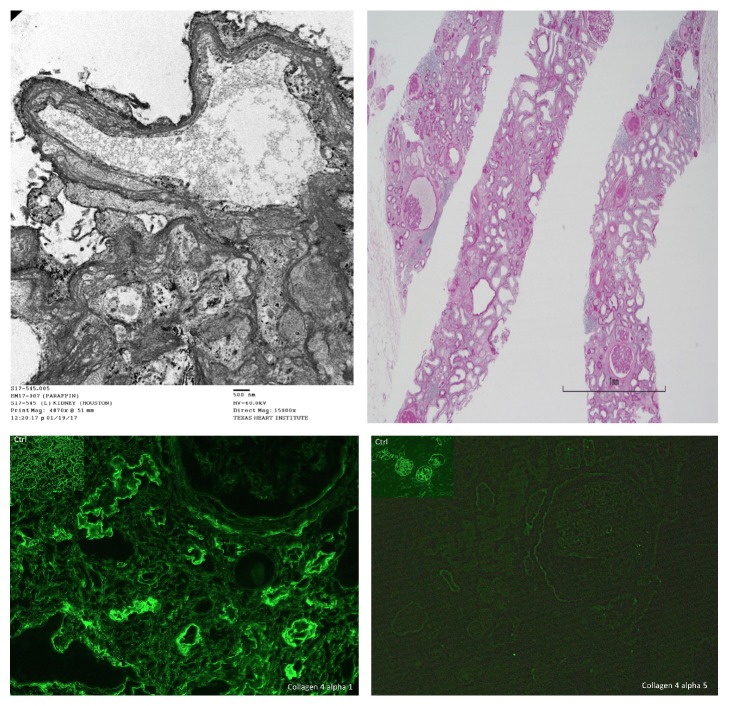
*Kidney Biopsy*. Electron microscopy identifying a split lamina densa (top left). Light microscopy demonstrating significant tubular atrophy and interstitial fibrosis (top right). Immunofluorescence highlighting absent staining for the a5 chain of type 5 collagen in the patient and no immune deposits (bottom row).

On Day 4 of the patient's hospitalization, the kidney biopsy revealed minimal inflammation and the absence of necrotizing vasculitis or crescent formation. The immunofluorescence did not show a “full house” pattern, despite high titers of both ANA and double-stranded DNA. Surprisingly, the electron microscopy revealed the characteristic features of Alport's syndrome (Figure 1). There was only 1 crescent out of 13 glomeruli, and no glomerular necrosis or immune deposition. The degree of interstitial fibrosis and tubular atrophy was 60%. There was loss of staining for alpha 3 and 5 chains of type 4 collagen. Due to the significant fibrosis and lack of active inflammation, both corticosteroids and PLEX were discontinued. Her renal function never recovered and she opted to pursue peritoneal dialysis.

## 2. Discussion

The rapidly declining renal function, double positive ANCA serologies, and recent initiation of Hydralazine led to the suspected clinical diagnosis of Drug-Induced ANCA Vasculitis (DIAV). Nearly 20 drugs have been reported to induce ANCA antibodies, but the more common culprits include Propylthiouracil, Hydralazine, Allopurinol, and Minocycline [1, 2]. The presence of antibodies directed against both MPO and PR3 antigens is not a common finding, but when present, it is highly suggestive of DIAV [3, 4]. The B-cells activated in DIAV can also produce other antibodies including anti-nuclear, anti-histone, anti-double-stranded DNA, anti-elastase, anti-lactoferrin, and anti-phospholipid antibodies [5]. Hence, the finding of multiple neutrophilic antibodies in a patient should raise suspicion for drug-induced injury. The classic kidney biopsy finding in DIAV is necrotizing or crescentic glomerulonephritis with a pauci-immune immunofluorescence. However, there are reports of patients with DIAV and minimal histologic changes on kidney biopsy [6]. Some patients have been shown to have immune complex deposition in contrast to the classic cases [6]. The role of these immune complexes is unclear. They may be directly pathogenic, a benign immune phenomenon induced by the drug or due to a secondary disease process all together.

DIAV has a better prognosis compared to primary ANCA Associated Vasculitis (AAV) [1]. The first line treatment is to remove the suspected culprit drug. If the biopsy shows severe inflammation or there is a severe decline in estimated GFR, then immunosuppressive therapy is warranted, though its use is largely based on published case reports. The Kidney Disease Improving Global Outcomes (KDIGO) guidelines recommend consideration of PLEX in patients with rapidly rising serum creatinine or diffuse alveolar hemorrhage. Importantly, the guidelines do not distinguish treatment of DIAV from primary AAV. Patients treated with plasma exchange (PLEX) were found to have lower incidence of end-stage kidney disease requiring dialysis, but there was no difference in mortality on long-term follow-up when compared to patients not treated with PLEX [7, 8]. A small study of 7 patients was able to show that plasma exchange decreased the levels of MPO and PR-3 antibody titers in all patients treated. However, the study was not designed to follow the patient's long-term clinical outcome. There is mixed evidence on whether MPO and PR-3 antibody titers correlate with disease severity or likelihood or relapse [9, 10].

Alport's syndrome can manifest with varying glomerular pathology including focal segmental, then diffuse glomerular sclerosis in an increasing number of glomeruli. There is no reported association between Alport's syndrome and necrotizing vasculitis. The hallmark lesion is diffuse thickening and splitting of the Glomerular Basement Membrane (GBM) with strikingly irregular outer and inner contours [11].

Alport syndrome (AS) is a hereditary disease of the GBM. Patients often present with hematuria, and it is associated with sensorineural deafness and ocular abnormalities. It accounts for 0.3 to 2.3% of all patients reaching end-stage kidney disease [11]. The disease is genetically heterogeneous and is associated with mutations in the genes:* COL4A3*,* COL4A4*, or* COL4A5*. These genes encode the *α*3(IV), *α*4(IV), and *α*5(IV) chains of type IV collagen, respectively [11, 12].

Genetic testing may be utilized in patients in whom the diagnosis of Alport's syndrome is suspected but biopsy results show nondiagnostic histologic findings. Genetic testing can provide prognostic information on the likelihood of progression to early renal failure or deafness based on the specific type of mutation identified. Genetic sequencing can also provide the patient with more information on the risk of his or her offspring inheriting the disease [13]. The decision to undergo genetic testing must be individualized because there is currently no disease-modifying treatment to alter the course of Alport's syndrome. Furthermore, test results have the potential to cause psychological harm [14]. The patient in this vignette was never previously treated with a RAAS inhibitor and elected not to pursue genetic testing.

The primary treatment for patients with Alport's is inhibition of the Renin-Angiotensin Aldosterone System (RAAS). Studies demonstrate that RAAS inhibition slows the progression of renal impairment even in patients without proteinuria [15]. Impaired activity of the nuclear 1 factor (erythroid-derived 2)–related factor 2 (Nrf2) transcription factor is implicated in chronic kidney disease by increasing oxidative stress and inflammation. The synthetic drug Bardoxolone Methyl activates the Nrf2 pathway and holds promise to suppress proinflammatory cytokine production [16]. The BEACON investigators studied Bardoxolone methyl in advanced diabetic kidney disease, but this study was terminated early due to increased cardiovascular events [17]. An ongoing clinical trial (CARDINAL) is underway to assess the safety and efficacy of Bardoxolone methyl in patients with Alport's syndrome.

## 3. Conclusion

To summarize, clinicians must be aware of the rare but serious consequence of drug-induced vasculitis in patients who are taking Hydralazine. When the decline in renal function is more rapid than expected, clinicians should search for multiple etiologies of kidney disease that may be superimposed on one another.

Alport's syndrome provided an explanation for our patient's chronic renal impairment. Clinicians must consider hereditary kidney disorders in patients with unexplained kidney disease and a positive family history. Because of the late diagnosis, there was a missed opportunity to intervene earlier with RAAS inhibitors.

A summary of our key teaching points can be found in Box 1.

## Figures and Tables

**Box 1:**
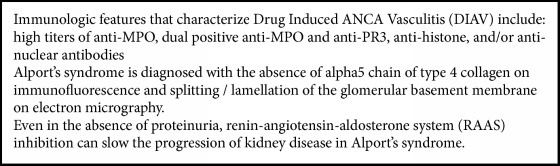
Key Teaching Points.

**Table 1:** Laboratory values on presentation.

	Patient values	Reference range
Sodium	150	135-148 meq/L
Potassium	4.9	3.6-5.5 meq/L
Total CO2	13	24-32 meq/L
BUN	189	10-26 mg/dL
Creatinine	16.66	0.50-1.2 mg/dL
Albumin	3.6	3.5-5 g/dL
Hemoglobin	5.8	12-15 g/dL
Anti-nuclear antibody	1:2,560	Negative
Anti-dsDNA	Negative	Negative
Anti-PR3	2.7 AI	<1 AI
Anti-MPO	>800 AI	<1 AI
Anti-histone	7.1 U	<1 U
Anti-glomerular basement Membrane Titer	Negative	Negative
Anti-streptolysin O	Negative	Negative
C3	71 mg/dL	82-193 mg/dL
C4	23 mg/dL	15-57 mg/dL
Serum electrophoresis	No monoclonal spike	
Urine drug screen	Negative	
